# A135 ENDOSCOPIC SUBMUCOSAL DISSECTION OF GASTRIC ADENOMAS AND EARLY CARCINOMAS

**DOI:** 10.1093/jcag/gwac036.135

**Published:** 2023-03-07

**Authors:** B Zhao, H J Kim, R Trasolini, D Chahal, E Lam

**Affiliations:** 1 Medicine; 2 Gastroenterology, University of British Columbia, Vancouver, Canada; 3 Gastroenterology, Harvard University, Cambridge, United States

## Abstract

**Background:**

Management of gastric adenoma and early gastric cancer requires endoscopic resection. This can often be achieved with endoscopic mucosal resection (EMR), which has been shown to be effective with a good safety profile. One disadvantage of EMR is that it is often completed piecemeal, leading to indeterminant margins and higher rates of recurrences that require additional intervention. Endoscopic submucosal dissection (ESD) is a more advanced endoscopic resection technique that has been shown to be more effective than EMR at en-bloc resection. ESD requires high technical proficiency but it is becoming more widely available in western countries.

**Purpose:**

The purpose of this study is to report on the outcomes and rates of complications of gastric ESD completed in a tertiary centre in British Columbia.

**Method:**

All gastric ESD was completed by a senior therapeutic endoscopist who has previously received training in Japan. Retrospective data were collected on all gastric ESD procedures done in St. Paul’s Hospital from May 7th, 2015, when the procedure first became available, to Aug 30th, 2022. Inclusion criteria were all adults who have undergone ESD for resection of a gastric lesion. Exclusion criteria were patients younger than 18. Data collected included demographic variables, polyp characteristics, procedural outcomes, and complications.

**Result(s):**

A total of 49 ESD procedures were completed. The mean size of the resected lesions was 25.3 mm (range: 5 – 100 mm). Technical success, defined as successful resection of all polypoid tissue, was achieved in 48/49 procedures (98.0%). En bloc resection was achieved in 42/48 (87.5%) completed ESD. The rate of R0 resection was also 42/48 (87.5%). Curative resection, defined as technically successful ESD with an R0 margin and no lymphovascular invasion, was achieved in 41/49 (83.7%) of the cases. In our cohort, 8 patients had adenocarcinoma, 5 of which had a curative resection with no evidence of recurrence. None of the ESD resulted in any intra-procedural or delayed perforation. 5/49 (10.2%) patients had clinically significant post-endoscopic resection bleeding. Out of 37 patients that completed follow-up, 3 (8.1%) had recurrence, and all of them were managed endoscopically. 4/49 (8.2%) of patients required surgery post-ESD.

**Conclusion(s):**

In our cohort, ESD is an effective endoscopic resection modality for gastric lesions with a high rate of technical success and curative resection. Despite a deeper plane of resection versus other endoscopic resection modalities, its complication rate remains low. Although ESD requires high technical proficiency, its favorable outcomes along with low rates of complication make ESD highly feasible for the resection of gastric lesions. Further research will be needed to study the implementation and outcomes of

ESD in a western setting.

**Please acknowledge all funding agencies by checking the applicable boxes below:**

None

**Disclosure of Interest:**

None Declared

